# Advancement in Electrospun Nanofibrous Membranes Modification and Their Application in Water Treatment

**DOI:** 10.3390/membranes3040266

**Published:** 2013-09-30

**Authors:** Shaik Anwar Ahamed Nabeela Nasreen, Subramanian Sundarrajan, Syed Abdulrahim Syed Nizar, Ramalingam Balamurugan, Seeram Ramakrishna

**Affiliations:** 1 NUS Nanoscience and Nanotechnology Institute, National University of Singapore, 2 Engineering Drive 3, 117581, Singapore; 2 Department of Mechanical Engineering, National University of Singapore, 2 Engineering Drive 3, 117575, Singapore

**Keywords:** electrospinning, nanofibers, synthesis, surface modification, interfacial polymerization, heavy metal, antibacterial

## Abstract

Water, among the most valuable natural resources available on earth, is under serious threat as a result of undesirable human activities: for example, marine dumping, atmospheric deposition, domestic, industrial and agricultural practices. Optimizing current methodologies and developing new and effective techniques to remove contaminants from water is the current focus of interest, in order to renew the available water resources. Materials like nanoparticles, polymers, and simple organic compounds, inorganic clay materials in the form of thin film, membrane or powder have been employed for water treatment. Among these materials, membrane technology plays a vital role in removal of contaminants due to its easy handling and high efficiency. Though many materials are under investigation, nanofibers driven membrane are more valuable and reliable. Synthetic methodologies applied over the modification of membrane and its applications in water treatment have been reviewed in this article.

## 1. Introduction

Nanoparticles, nanofibers and other nanostructures bring tremendous technological advancements in the field of electronics [1], catalysis [2], bioengineering [3] and environmental applications [4]. Such nanostructures using various materials such as polymeric [5], inorganic metal/polymer composite [6] with variations in their composition, configuration and assembly have been produced by various techniques such as electrospinning [7], template assisted synthesis [8], phase separation [9], self assembly [10], solvent evaporation [11], drawing-processing method [12] and doctor blading method [13]. Among them, electrospinning is one of the most versatile and simple techniques, which has been applied to fabricate one dimensional nanostructures viz., nanofibers. Recently, numerous journal articles have documented electrospun nanofibrous “Membranes” (ENMs) for water treatment applications. A review article by Balamurugan *et al.* [14] reports on recent trends in nanofibrous membranes and their suitability for air and water filtration applications, preparation and characterization of electrospun nanofibers membranes and their possible applications in water treatment. Feng *et al.* [15] and Subramanian *et al.* [16] report on “New Directions of nanofibers in nanofiltration applications”. These are some of very recent reports which emphasize the importance of ENMs in water technology.

ENMs have unique and interesting features, such as high surface area to volume ratio, large porosity, good mechanical properties and good water permeability, which provides a major contribution towards water treatment. These nanofibers were employed for the various water treatment applications based on their thickness, porosity, and surface roughness. The most widely applied filtration methods are microfiltration [17], ultrafiltration [18], nanofiltration [19], reverse osmosis [20] and forward osmosis and pressure retarded osmosis [21]. Among them, electrospun nanofiber membranes were exploited for the first three applications, which are covered in this review.

## 2. Nanofiber Preparation—Electrospinning Technique

**Figure 1:**
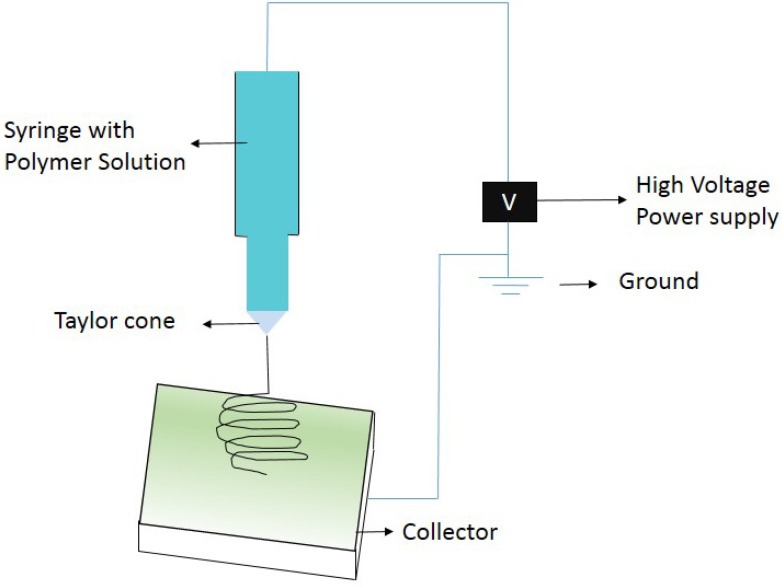
Electrospinning set up.

Electrospinning is a versatile technique for manufacturing of nanofibers with different diameter and varied morphologies. Ultrafine nanofibers from micro to nano scale can be produced with ease. Solution viscosity, applied voltage range, humidity, tip to the collector distance are some of the important parameters that governs the formation of nanofibers. Variations in the above mentioned parameters will result in formation of thin to thick nanofibers and smooth to corrugated surfaces. A simple diagram of the electrospinning set up is shown in Figure 1.

In this process, a high voltage is applied to create an electrically charged jet of polymer solution from a syringe. The voltage is applied gradually and when the applied voltage overcomes the surface tension of the polymer solution, a Taylor cone appears and it spins down as a fiber to reach the collector plate. Before reaching the collector, the solvent evaporates and the polymer solidifies and gets collected as fibers. One end of the supply is connected to a syringe needle and the other to the collector that is grounded. Solution from the jet which is held by the surface tension will overcome once induces a charge on the surface of the liquid. Repulsion and contraction of the surface charges to the counter electrode cause a force directly opposite to the surface tension. As the voltage increased, the Taylor cone formed from the tip of needle surface elongates, *i.e.*, the fluid elongates to attain a critical value where the repulsive force overcomes the surface tension and discharge. The discharged polymer solution jet undergoes an instability and elongation process, which allows the jet to become very long, uniform and thin fibers. 

Although electrospinning is a methodology with numerous potential in various applications, one of the main disadvantages is the disability to achieve large scale productivity. Recently, multijet [22] and needless electrospinning techniques [23] are emerging to bridge the gap of large scale manufacturing.

## 3. Modifications of Electrospun Nanofiber Membranes (ENMs)

### 3.1. Surface Modification of ENMs

Electrospun nanofibers are being synthesized at an increasing rate to meet its demand for various applications. Many different methods of synthesis of polymer for electrospinning to the targeted applications are available. The established methods include sol-gel synthesis [24], *in situ* polymerization [25], surface modification [26], plasma induced grafting [27], Graft polymerization [28], blending [29], polymer-inorganic composites formation techniques [30], *etc.* The resultant physical morphology and mechanical properties vary depending on the polymeric concentration and spinning condition employed during the process. Selectivity in contaminant removal, mechanical strength, and porosity of the polymer network can be modified in the ENMs in order to improve their performances towards water purification. The surface modifications of the ENMs enhance the nanofibers matrix properties such as availability of functional groups on the surface of nanofibers. Some of the other surface modification techniques are oxidation process [31], plasma treatment [32], solvent vapor treatment [33] and surface coating [34].

**Figure 2:**
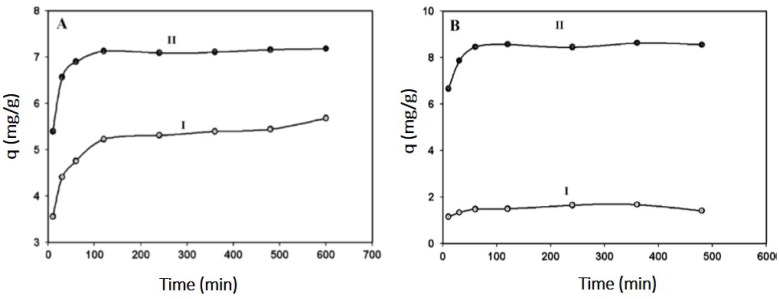
Effect of contact time on adsorption: (**A**) Pb and (**B**) Cd (I: cellulose and II: cellulose-g-oxolane-2,5-dione nanofibers). (Reprinted with permission from [35]. Copyright 2011 Elsevier).

Cellulose, a biopolymer made nanofibers is commonly used as adsorbent for the water filtration studies. The optimal required capacity of the cellulose fiber was not attainable, due to its low surface area and stability. Synthetic modifications of the cellulose materials with organic functional groups are very important in order to improve the polymers activity with the pollutant either by adsorption or sensing. Stephen *et al.* [35] modified nanofibers with oxolone-2,5-dione, which not only enhanced the surface area of the nanofiber mat and also helped in detecting heavy metals like cadmium and lead. The adsorption capacities based on time studies of these membranes (Figure 2) were compared with commercial adsorbents such as Dowex and Amberlite resin. They also suggested that these modified nanofibers membranes can be regenerated by treating with nitric acid and reused.

The modification of chitosan fibers was conducted by Schiffman *et al.* by crosslinking [36] using Glutaratldehyde and shciffs imine. Followed by Schiffman, Haider *et al.* [37] reported the solubility of chitosan nanofibers by treatment with trifluoroacetic acid (TFA). TFA forms a salt and exists in the form of ammonium cation and trifluoroacetate anion in the fibers. The amine group of chitosan was made available when this nanofiber was subjected to base treatment with K_2_CO_3_. The potassium cation binds with acetate and undergoes neutralization. This neutralization has allowed free amine group, which preferred to absorb more heavy metals. The salts helped the nanofiber mats to remain stable in aqueous medium to remove the contaminants. They reported that Cu (II) adsorption of nanofibers were ~6 and ~11 times higher than chitosan microsphere (80.71 mg/g) and the plain chitosan (45.20 mg/g), respectively.

**Scheme 1:**
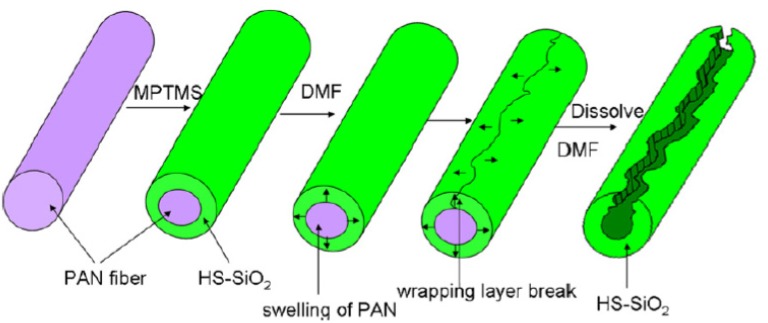
Fabrication procedure of zonal mercaptopropyl silica nanofibers obtained by dissolution of the PAN nanofiber templates with DMF. (Reprinted with permission from [38]. Copyright 2011 Elsevier).

Nanofiber template assisted synthesis of Silica nanofibers were studied by Li *et al.* [38] for the removal of heavy metals like mercury from waste water. Mixture of ethanol, HCl, (3-mercaptopropyl) trimethoxysilane were hydrolyzed and coated onto the PAN nanofibers template. After drying, the nanofibers template was removed by dissolving the PAN in DMF to produce zonal mercaptopropyl silica (ZMS) nanofibers (Scheme 1). The maximum adsorption capacity exhibited for ZMS nanofibers within 60 min of contact time was 57.49 mg/g compared to pure silica nanofibers of 1.36 mg/g.

**Scheme 2:**
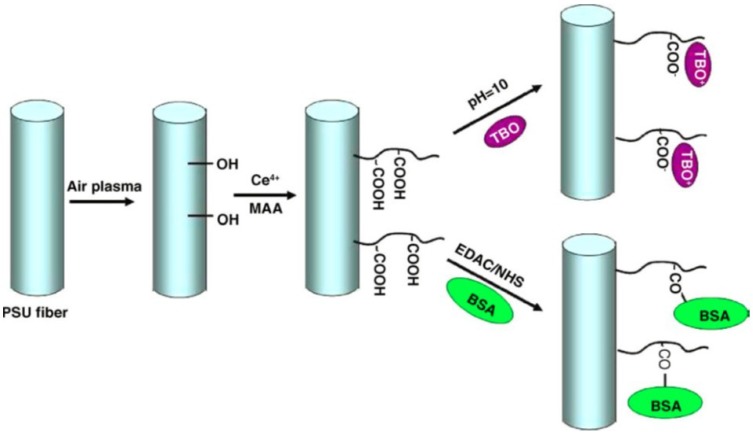
Schematic diagram of the surface modification process of the electrospun PSU fiber. Followed by the absorption of TBO. (Reprinted with permission from [40]. Copyright 2006 Elsevier).

*In situ* polymerization of fluorinated polybenzoxazine layer (F-PBZ) incorporated with silica nanoparticle was carried out on the cellulose acetate nanofibers surface by Shang *et al.* [39] These modified nanofiber exhibited superhydrophobicity with the water contact angle of 161° and superoleophilicity of 3°. This membrane showed an excellent separation of oil-water mixtures and also worked stable with wider pH range (2–14) suggest that they can be used for practical oil-polluted water treatments and oil spill cleanup. Ma *et al.* [40] reported on the fabrication of polysulfone nanofibers and its modifications with MAA (methacrylic acid) by grafting technique (Scheme 2). They treated PSU nanofibers to air plasma followed by immersing nanofibers in the solution of methacrylic acid to form PMAA grafted PSU nanofibers membrane. Toluidine blue O (TBO dye) were removed using these nanofibers and their adsorption capacity was ~380 nmol/mg. Protein ligands (BSA) were covalently functionalized and immobilized on the PMMA grafted PSU membranes. These ENMs showed lower pressure drop and high flux (2 mL/cm^2^ min) compared to the conventional membrane (5–20 psi).

Yoon *et al.* [41] reported on the modification of poly (ether sulfone) (PES) nanofiber by adding mixed solvents (DMF: NMP) to improve the mechanical properties and oxidation process. The hydrophilicity of the membranes were improved by treatment of the nanofibers with 3% *w*/*v* of ammonium per sulfate, whereas improvement in mechanical strength of modulus and strength 570% and 360% respectively were observed by the addition of high boiling solvent NMP with DMF (50%:50%) The hydrophilicity values for the untreated and treated membranes were found to be 120° and 28° respectively. Silver and silver ions have been widely used as an antimicrobial agent. The incorporation of silver ions into nanofibrous membranes by electrospinning is an attractive method to fabricate nanofibers having the ability to remove pathogen and suspended particle from waste water. Biorge *et al.* [42] used different polymer membranes immersed in AgNO_3_ followed by NaBH_4_ reduction, which resulted in the formation of silver ions, which acted as an antibacterial agent and inhibits/disrupts the bacterial cell. The clean water permeability (CWP) was also quite high for these membranes.

**Figure 3:**
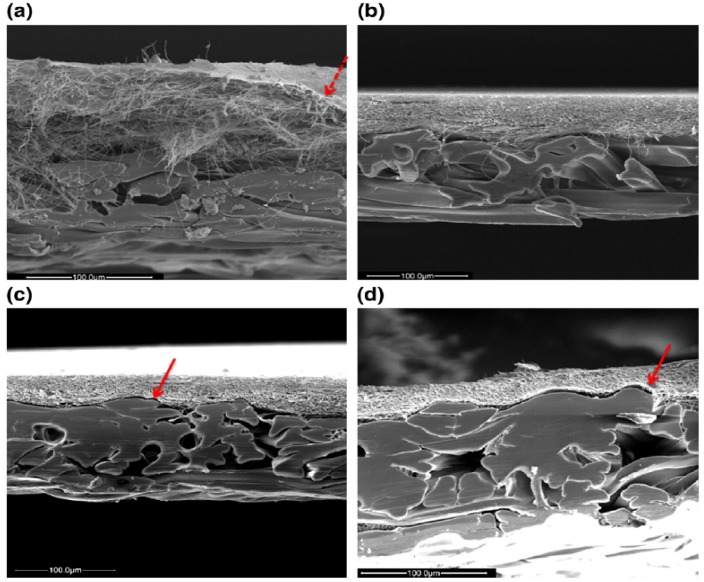
Thickness of (**a**) electrospun nanofibrous membranes (ENM)-control; (**b**) ENM-1; (**c**) ENM-2 and (**d**) ENM-3. (Reprinted with permission from [44]. Copyright 2011 Elsevier).

Microporous membranes were produced by Li *et al.* [43] to control the pore size of the electrospun membrane by annealing. Pore size reduction from 2.8 to 0.9 µm with reduced porosity were observed when annealed at different temperatures (90–105 °C) and at different time intervals (30–120 min). The tensile strength was increased about 8 fold from 15.4 to 126 MPa with a major change in the contact angle. These nanofiber membranes efficiently remove TiO_2_ particles. Furthermore, hot pressing of nanofibers with different pressure value has enhanced the membrane performance in particle rejection [44]. An applied pressure of 0.14 Mpa has reduced the bubble point of the hot pressed membrane and further increasing the pressure has resulted in drastic decrease in the bubble point. The SEM images of the hot pressed membrane were represented in Figure 3. 

Figure 3a represents the loose structure of electrospun nanofibrous membranes before hot pressing and it can be easily compressible after hot press (dotted arrow on top layer) as shown in Figure 3b–d indicates the well-organized structure after hot pressing other nanofibers membrane (arrow indicated the top layer after hot press).

Nitrile group of polyacrylonitrile (PAN) nanofiber membranes were reduced to amino groups and coupling of hydrophilic flexible spaces followed by reaction with poly hexamethylene guanidine hydrochloride (PHGH) were carried out by Mei *et al.* [45] the spacer groups have improved the hydrophilicity of the membrane and guanidine hydrochloride acted as an antibacterial agent. The resulting PHGH immobilized nanofiber membranes exhibited highly effective antibacterial activities even after 3 cycles of antibacterial assays. The pure water flux of unmodified and modified electrospun nanofibers were measured using dead end filtration method which include PAN, PAN–NH_2_, PAN–NH_2_–GDGE (spacer group)–PHGH (antibacterial agent), and PAN–NH_2_–PEGDGE (spacer group)–PHGH (antibacterial agent) had average water flux of 15,515, 16,194, 26,276, and 30,009 L/m^2^ h, respectively. All these surface modifications were summarized in Table 1.

**Table 1:** Nanofiber-surface modification.

S. No	Material	Modification	Active group	Target metal	Removal	Ref.
1	chitosan	neutralization with K_2_CO_3_	–NH_2_–, amine	Cu(II)	485.44 mg/g	[37]
				Pb(II)	263.15 mg/g	
2	silica	zonal dissolution of PAN	–SH–, Thiol	Hg(II)	57.49 mg/g	[38]
3	cellulose acetate	*In situ* polymerization	fluorinated polybenzoxazine	oil water	maximum	[39]
4	poly sulfone	graft copolymerization	carboxyl group	toluidine blue O,BSA	380 nmol of TBO/mg of TBO	[40]
5	poly ether sulfone	1. solvent induced fusion	carbonyl	waste water	1. flux: 2626 L/m^2^h psi	[41]
		2. oxidation			2. flux: 2913 L/m^2^h psi	
6	PETE, PCTE, PTFC, PA	AgNO_3_ reduction	Ag	pathogen, waste water	turbidity removal: 99.25%	[42]
					COD: 94.73%	
					NH4^+^: 93.98%	
7	poly lactic acid	annealing	–COOH–	TiO_2_ removal	85% rejection	[43]
8	polyacrylo nitrile	hot press interfacial polymerization	–CN–	salt rejection MgSO_4_	86.5%	[44]
9	polyacrylo nitrile	coupling	–NH_2_–	antibacterial	53.7%–99.9%	[45]

Zhao *et al.* [46] applied the coating of polymer solutions as a barrier layer on ENMs to enhance the performance of ultrafiltration or nanofiltration medium. Chitosan, modified chitosan with glutaraldehyde, terephthaloyl chloride were tested. The modified PVDF membranes showed good flux rate and rejection efficiency to bovine serum albumin filtration at 0.2 MPa. The flux of 70.5 L/m^2^ h, rejection efficiency of >98% were reported for the ENMs when compared to the commercially available UF membranes.

### 3.2. Interfacial Polymerization

The main application of the interfacial polymerization on the electrospun nanofibrous materials is nanofiltration. Nanofiltration technique uses thin film composite membrane media for the removal of salts from the brackish water and sea water. These membranes have the basic configuration of (1) top ultrathin selective barrier layer; (2) middle porous support membrane and (3) bottom non-woven fabric to maintain strength of the whole configuration. Layers 1 and 2 can be fine tuned in order to control the performances of the membrane.

Various parameters have to be taken into consideration in the interfacial polymerization, such as reactant concentration, the partition coefficient of the reactant, the reactivity ratio, kinetics and diffusion and processing procedure [47].

Many reports have documented the modifications of the barrier layer on top of the porous polymer membrane. Yoon *et al.* [48] reported the interfacial polymerization at three different ratios of piperazine and bipiperidine on PAN nanofiber membranes. The study involves the rejection of divalent MgSO_4_ (2000 ppm) using cross flow technique. The rejection rate was improved when the concentration of Piperazine increased from 0.25% to 1% but the permeation reduced due to the thick barrier layer formation (>95% removal of salt), at pressure range of 70–190 psi. This was achieved by monomer solution with permeate flux 2.4 times greater than the TFC membrane.

Modification in the barrier layer enhances the water treatment properties. Changes in the additives to the barrier layer and heat treatment to the support membrane improve to the permeation flux 2 to 3 times higher. The same methodology of IP was carried out by Yung *et al.* [49] with PES support membranes. Ionic liquids (IL) were added as the additives to the barrier layer, which acts as a substitute to organic solvents. The ILs did not contribute to the polymerization process; rather, they worked between the surfactant and ionic salt to vary the aqueous phase during interfacial polymerization. The addition of IL tightens the crosslinking polyamide barrier layer by propagating the polymerization process and it leads to 138% improved rejection of NaCl when compared to non IL treated membranes (24.3% NaCl).

Wu *et al.* [50] reported the modifications of interfacial polymerization using B-cyclodextrin (CD). Trimesoyl chloride, triethanolamine and CD were used as the interfacial polymerization additives. Concentration of 1.8% (*w*/*v*) of CD in aqueous phase shows a 2 fold increase in the value of water flux of TFNC than normal polyester membranes. This TFNC indicates remarkable increase in salt rejection and surface charge. The antifouling properties of this TFNC had been discussed in this research article. The higher hydroxyl content on CD inhibits the crosslinking reaction and leads to strong intermolecular hydrogen bonding which results in smoother membrane surface.

### 3.3. Other Modifications

Blending of PVDF with surface modified macromolecules were studied by Kaur *et al.* [51] and the macromolecules were synthesized separately using urethane prepolymer with different molecular weighted polyethylene glycols (PEGs) and these blends shows significant improvement in the hydrophilicity of the blend fibers. Poly (vinylidenefluoride) (PVDF) when blended with clay nanocomposites the hydrophobicity of the membrane increases in the mixture. The highest water contact angle achieved was 154.20° ± 3.04° and melting point of the PVDF–clay electrospun nanofiber membrane increases with the increasing concentration of clay. These increments in the melting point indicates clay’s role in the crystallization process of the nanocomposite membrane [52]. Electrospinning followed by surface modification of PVDF nanofibers membrane produces superhydrophobic membranes. The modification includes dopamine surface activation, silver nanoparticle deposition and hydrophobic treatment. These unmodified and modified membrane (I-PVDF) can achieve a high and stable water flux of 31.6 L/m^2^ h using a 3.5 wt % NaCl as the feed solution while the feed and permeate temperatures were fixed at 333 K and 293 K, respectively [53].

A high flux thin film nanofibrous composite (TFNC) membrane based on PAN nanofibers coupled with thin barrier layer of cross linked poly vinyl alcohol. With a middle-layer PAN scaffold with porosity of 85% and cross-linked PVA barrier layer with thickness of about 0.5 µm, the TFNC membrane system were tested for ultrafiltration (UF) applications. These material exhibits a very high flux up to 12 times higher than that of conventional PAN UF membranes and excellent rejection ratio of (>99.5%) for separation of oil/water mixture of 1500 ppm in water over a long time period (tested up to 190 h) at pressure range up to 130 psig [54].

Poly (vinyl alcohol) (PVA)/polyacrylonitrile (PAN) nanofibrous composite membranes were prepared by electrospinning. PVA nanofiber layers were spun thicknesses of several micrometers on the electrospun PAN nanofibrous substrate. The spun PVA nanofibers were melted by water vapor to form a thin film of barrier layer and chemical crosslinking in glutaraldehyde water/acetone solution. Highest permeate flux of 210 L/m^2^ h was achieved with the rejection of 99.5% for the membrane under the operating pressure of 0.3 MPa. This methodology opens a way to fabricate membranes with other polymeric materials and its treatment with suitable solvent vapour to form TFNC membranes [33]. The same methodology was handled by Huang *et al.* [55], where a post-treatment approach was demonstrated to improve the mechanical properties of polymers like polyacylonitrile (PAN) and polysulfone (PSu). The mechanical strength of this polymer membrane was improved by the solvent-induced fusion of inter-fiber junction points. The treated membranes showed significant enhancement on tensile strength and Young’s Modulus while high porosity and water permeability were retained.

Polyamide made Reverse Osmosis membranes were modified by free-radical graft polymerization of 3-allyl-5,5-dimethylhydantoin (ADMH) using 2,2-azobis (isobutyramidine) dihydrochloride as an initiator. The water flux of the ADMH-grafted membranes was higher with slightly decreased salt rejections. The chlorine resistances of the ADMH-grafted membranes were significantly improved when compared to the raw membrane [56]. PVDF nanofibers membrane was modified by grafting acrylic acid and meth acrylic acid both by chemically and plasma. High water flux of 150 kg/h m^2^ at an operating pressure of 4 psig, and a 79% removal of polyethylene oxide (molecular weight 400 kDa) were achieved [57].

## 4. Application of ENMs in Water Treatment

### 4.1. Heavy Metal Removal

Heavy metals generated by industries, indiscriminate human activities, *etc.*, can cause serious damages to both environment and human health. These heavy metals are quite often mixed with water resources and are also well distributed in the entire environment. Removal of these pollutants from water resources is a major concern and many researches are focusing to address this issue. Nanofibers play a major role among these available purification/removal techniques. Few such pollutants and its removal by nanofibers will be discussed in this section.

Among the heavy metal ions released into the environment, chromium is considered as a primary toxic pollutant in water resources. The hexavalent chromium poses serious threats to human by causing cancer. Many technologies and materials have been employed and nanofiber membrane technology is preferable due to its remarkable characteristics like large porosity and surface area as mentioned before. Individual polymer or composites exhibits tremendous performances against chromium removal. Taha *et al.* [58] reported the synthesis of amine functionalized cellulose acetate/silica composite nanofiber membranes. The amine functionalized nanofibers, due to the electrostatic interaction/chelation process, enables chromium(VI) adsorption and removal and were quantized to be 19.45 mg/g. Removal of Cr(II) up to 97 mg/g by changing the polymer matrix from CA to PVA have also been reported by same group [59] The smaller diffusion resistance of Cr^3+^ leads to easy entry and easy binding with the mesoporous membrane.

Composites membranes of PAN/FeCl_3_ exhibit about 110 mg/Cr g removal and converts Cr(IV) to Cr(III), which is less harmful. The mechanism of the nanofibers membrane process is as follows [60]:

PAN–Fe(II)OH^+^ + Cr_3_O_7_^2−^ → PAN–Fe*_x_*(III)Cr(III)(OH)_3_ + H^+^

PAN–Fe(II)OH^+^ + Cr_2_O_7_^2−^ → PAN–Fe(III)*_x_*Cr(III)*_x_*(OH)_2_ + H^+^

Increase in the FeCl_3_ content reduces the adsorption of chromium and the excess iron will diffuse into the solution and reacts with chromium reducing its concentration in solution. In relation to this data, Li *et al.* [61] reported the composite membrane made of polyamide 6 and Fe*_x_*O*_y_*. The adsorption capacity of 150 mg/g was reported which is higher than the previously reported values for Cr(VI) removal. The formation of Fe nanoparticles and its protonated form helps in adsorption/reduction of Cr(VI) to Cr(III). 

HCrO^4−^ + 3Fe^2+^ + 7H^+^ → Cr^3+^ + 3Fe^3+^ + 4H_2_O

Xu *et al.* [62] reported the hierarchical growth of nanofiber membranes using thermo plastic elastomeric ester (TPEE) and Iron oxide. Copper and lead were removed by chitosan nanofibers mats [24]. The removal of these metal ions was either by chelation/electrostatic adsorption method and the adsorption capacity is reported to be 485.44 mg/Cu g and 263.15 mg/Pb g, which is six times greater than the previously reported values.

Removal of other toxic metal ions like nickel, cadmium along with copper and lead have been reported by Aliabadi *et al.* [63] Composition of PEO and chitosan nanofiber membranes was employed to study the purification process. The mesoporous composite nanofibers exhibit 183.4 mg/Ni g, 172.3 mg/Cu g, 150.3 mg/Cd g, 143.4 mg/Pb g for a pseudo first order model and 249.9 mg/Ni g, 229.2 mg/Cu g, 196.6 mg/Cd g and 195.1 mg/Pb g for second order removal from the solution.

### 4.2. Microbial Removal

An atmospheric helium plasma treatment was employed to reduce AgNO_3_ to Ag nanoparticles. This prepolymer solution with PAN electrospun to form nanofibers membranes with Ag nanoparticles sized in the range of 3 to 6 nm. Gram positive *Basillus cereus* and gram negative *Escherichia coli* microorganisms were tested using this fiber. The fibers without silver compounds ended with no antibacterial activity compared to the Ag nanoparticle doped PAN nanofibers [64]. The same methodology was applied to PAN nanofibers by amidoxime functionalized PAN. Coordination of Ag^+^ and its reduction to Ag nanoparticles were tested for microbes like S.aurieus and *E. coli*. The ASFPAN-3, which were amidoxime functionalized nanofibers after immersion for 20 min in NH_4_OH showed log 7 reductions (complete kill). The amidoxime group tends to bind with metal ions like Mg^2+^ and Ca^2+^, which are essential for the bacterial stability and replication through co-ordination. The competency of the amidoxime coordination with bacterial holding increased and more metals bind to amidoxime group rather on to the cell membrane of the bacterial cell. This process restricts the cellular replication and growth of the bacteria and kills the cell. The same trend was observed for both AgNO_3_ solution dipped nanofibers membrane for 30 min and Ag nanoparticle/PAN nanofibers, exhibits log 7 reduction bacteria [65]. Few other reports using nanofiber mats were tabulated in Table 2.

**Table 2:** Nanofibers for removal of bacteria.

Polymer	Membrane diameter (nm)	Properties					Antibacterial activity	Ref.
Poly acrylonitrile (PAN)	100	Mean Pore Size: 0.22 ± 0.01 µm			Flux: 1.5 L/m^2^h		*E. coli*	[66]
Polyacrylonitrile (PAN)	50	Mean Pore Size: 0.4 µm			–		*S. aureus*	[67]
							*E. coli*	
Nylon-6	650	OD culture at 600 nm					*S. aureus* *E. coli*	[68,69]
		*E. coli*			Pristine-3.4			
					Mat 1-1.57			
					Mat 2-1.75			
		*S. aureus*			Pristine-2.55			
					Mat 1-1.68			
					Mat 2-1.88			
Polyacrylonitrile (PAN)	200	Zone inhibition (mm)					*B. subtiliss* *S. aureus* *E. coli*	[70]
		Microorganism	NaBH_4_ reduction	Heated @160 °C		Heated @80°C		
		*B. subtilis*	7.5	6		10		
		*S. aureus*	9	10		10		
		*E. coli*	–	6		9		

### 4.3. Desalination

Desalination an effective technology for overcoming the higher demand for water. Various desalination technologies have been developed, which include reverse osmosis (RO), membrane distillation (MD), freeze desalination (FD), electrodialysis (ED), ion exchange (IX), and nanofiltration (NF). Among them, NF technology has been emerging as one of the effective ones to desalt low salt content water due to enhanced flux, lower operational pressure and energy savings. Nanofibers are currently explored as potential membranes for desalination due to their improved flux performance. A comprehensive listing of ENMs used in desalination applications and their performances are presented in Table 3.

**Table 3:** Electrospun nanofibers in the desalination application.

Middle layer (electrospun nanofiber)	Third layer	Solute	Method	Flux (L/m^2^/h)	Rejection (%)	Ref.
PVA/MWNT or Pebax/MWNT over PET substrate	none	oil/water	TFNC by coating	330 or 160	n.a.	[71]
PVA or Pebax over PET substrate	none	oil/water	TFNC by coating	130 or 58	PVA coated >99.5	[72]
10 and 4 wt % of PAN over PET substrate, rotating collector	none	oil/water	TFNC by coating	TFNC an order of magnitude > com.	99.5%, better than com. NF	[73]
PAN	polyamides	MgSO_4_	TFNC by Interfacial	TFNC 38% > com. NF 270	TFNC and com. are comparable	[49]
PVDF	polyamides	MgSO_4_	TFNC by Interfacial	0.66	75.7	[74]
		NaCl		0.66	70.2	
PAN	polyamides	MgSO_4_	Interfacial			[45]
			TFNC1	–	88	
			TFNC2	81	84.2	
first layer 8 or 10 wt % PAN	polyamides	MgSO_4_	Interfacial	220	89	[75]
second layer 4 or 6 or 8 wt % PAN		NaCl		200	89	
PVDF	n.a.	6 wt % NaCl	AGMD	11–12 kg/(m^2^ h)	n.a.	[76]
PVDF	n.a.	NaCl	DCMD	n.a.	98.27	[52]
PVDF-clay nanocomposites					99.95	
PET/PS	polyamide	NaCl	Interfacial	1.13 L m^−^^2^ h^−^^1^ bar^−^^1^	–	[21]

Notes: n.a.: not available; com.: Commercial membranes; AGMD: air gap membrane distillation; DCMD: direct contact membrane distillation.

The conventional middle layer was replaced with ENMs and then coating with various materials was carried out to form thin film nanocomposite (TFNC) membranes by Chu group [71,72,73]. They observed that the flux rate and oil rejection (oil in water emulsion) of the TFNC membranes was higher than commercial NF 270 membranes [72]. The thin layer formation through the interfacial polymerization technique by the reaction of polyamines in water with polyacid chlorides in organic solvents was also carried out by Chu and Ramakrishna group. The ENMs were also used as self-supporting membranes for the desalination application by Kaur *et al.* [77] The ENMs were also explored for membrane distillation application by Ramakrishna group [75]. The ENMs were stable up to 25 days tested and these ENMs may compete with conventional distillation and RO processes. Blending of clay nanoparticles with PVDF followed by electrospinning was carried out for direct contact membrane distillation (DCMD) process and greater than 99.95% salt rejection was achieved by Prince *et al.* [52]. 

The PAN based carbon nanofibers (CNFs) were also explored for the capacitive deionization by Wang *et al.* [76]. They have observed higher electrosorption capacity (4.64 mg/g) for CNFs than other materials (activated carbon (3.68), woven carbon fibers (1.87), carbon aerogel (3.33), CNTs-CNFs (3.32), mesoporous carbon (0.69), and graphene of 1.85 mg/g), which shows that ENMs can be potentially applied in electrochemical capacitive deionization of seawater desalination.

### 4.4. Other Application

Novel crosslinking chemistry can also be introduced into the nanofiber to improve the filtration efficiency effectively since the fiber diameter of nanofibers can be fine tuned to get smaller pore diameter than meltblown and spunbound layers for better performance of the later. This idea has been applied in the crosslinking of PVA nanofibers by maleic acid using vitriolic acid as a catalyst to improve the antiwater properties by Qin *et al.* [78] The filtration efficiencies of the melt blown and spunbound sublayers were, 30% and 6%, respectively, whereas the filtration efficiency of complex is much higher than those sublayers after 0.5 g/m^2^ crosslinked nanofibers membrane was electrospun on the sublayers. When 1.9 g/m^2^ and 2.9 g/m^2^ nanofibers webs were electrospun on the meltblown sublayers, and spunbonded sublayers, the filtration efficiencies of about 100%, and 95% were observed for meltblown complex, and spunbonded complex, respectively.

The applicability of polyethersulphone ENMs supported on a PET sub-layer for liquid filtration was studied by Homaeigohar *et al.* [79]. The ENMs showed high permeability for the pure water flux and at high feed pressures the water permeation slowly decreased. They applied heat treatment approach to overcome the stability of these fibers and particles of size >1 µm were removed in an hour with very high flux and low pressure. When they tested for a feed containing nanoparticles (<1 µm size), the major rejection was achieved within the first hour due to pore blocking.

A novel double layer PAN nanofiber membrane was reinforced with 2,2,6,6-tetramethylpiperidine-1-oxyl (TEMPO) by Cao *et al.* [80] in which radical oxidizes jute cellulose nanowhiskers. These PAN/cellulose composite nanofibers showed good mechanical strength and high filtration efficiency for 7–10 nm particles. These nanofibers can be extended for application in the domestic drinking water and industrial waste water treatment. 

## 5. Future Directions and Conclusions

Various synthetic methodologies such as *in situ* polymerization, addition of molecular dopants, inter or intermolecular bondings in combination with electrospinning techniques may be pursued in future to produce nanofibers with different morphologies and their filtration performances. The surface modification by grafting, interfacial polymerization, and nanoparticles coating, treatment with acids are found to be suitable methods to alter the surface properties, improve the filtration performance and antifouling properties of nanofiber membranes. In the case of heavy metal ion removal, the adsorption capacities of such modified membranes were comparable/better than conventional membranes/commercial agents. The electrospun nanofibers were also effectively used in the filtration of oil/water emulsions and microparticles.

The electrospun nanofibers can also be potentially applied in the areas of nanofiltration, membrane distillation, geothermal water desalination and capacitive deionization applications. The hot pressing step is necessary to improve the integrity of the nanofiber membranes as well as nanofiber/backing layer in the case of TFNC membranes in NF application. When the middle layer of conventional membranes was replaced with nanofibrous membranes, improved flux than conventional membranes was observed.
